# Development and Feasibility of a Family-Based Health Behavior Intervention Using Intelligent Personal Assistants: Randomized Controlled Trial

**DOI:** 10.2196/17501

**Published:** 2021-01-28

**Authors:** Angela Carlin, Caomhan Logue, Jonathan Flynn, Marie H Murphy, Alison M Gallagher

**Affiliations:** 1 Centre for Exercise Medicine, Physical Activity and Health Sport and Exercise Sciences Research Institute Ulster University Newtownabbey United Kingdom; 2 Nutrition Innovation Centre for Food and Health Biomedical Sciences Research Institute Ulster University Coleraine United Kingdom

**Keywords:** children, parent, physical activity, healthy eating, technology, mobile phone

## Abstract

**Background:**

Intelligent personal assistants such as Amazon Echo and Google Home have become increasingly integrated into the home setting and, therefore, may facilitate behavior change via novel interactions or as an adjunct to conventional interventions. However, little is currently known about their potential role in this context.

**Objective:**

This feasibility study aims to develop the Intelligent Personal Assistant Project (IPAP) and assess the acceptability and feasibility of this technology for promoting and maintaining physical activity and other health-related behaviors in both parents and children.

**Methods:**

This pilot feasibility study was conducted in 2 phases. For phase 1, families who were attending a community-based weight management project were invited to participate, whereas phase 2 recruited families not currently receiving any additional intervention. Families were randomly allocated to either the intervention group (received a smart speaker for use in the family home) or the control group. The IPAP intervention aimed to promote positive health behaviors in the family setting through utilization of the functions of a smart speaker and its linked intelligent personal assistant. Data were collected on recruitment, retention, outcome measures, intervention acceptability, device interactions, and usage.

**Results:**

In total, 26 families with at least one child aged 5 to 12 years were recruited, with 23 families retained at follow-up. Across phase 1 of the intervention, families interacted with the intelligent personal assistant a total of 65 times. Although device interactions across phase 2 of the intervention were much higher (312 times), only 10.9% (34/312) of interactions were coded as relevant (related to diet, physical activity or well-being). Focus groups highlighted that the families found the devices acceptable and easy to use and felt that the prompts or reminders were useful in prompting healthier behaviors. Some further intervention refinements in relation to the timing of prompts and integrating feedback alongside the devices were suggested by families.

**Conclusions:**

Using intelligent personal assistants to deliver health-related messages and information within the home is feasible, with high levels of engagement reported by participating families. This novel feasibility study highlights important methodological considerations that should inform future trials testing the effectiveness of intelligent personal assistants in promoting positive health-related behaviors.

**Trial Registration:**

ISRCTN Registry ISRCTN16792534; http://www.isrctn.com/ISRCTN16792534

## Introduction

The high incidence of childhood obesity has been well documented, with 29% of children aged 2 to 15 years in England [1] and one-fourth of children living in Northern Ireland [2] classified as overweight or obese. Furthermore, approximately one-fifth of children in the United Kingdom meet the recommended guideline of at least 60 minutes per day of moderate-to-vigorous physical activity [3,4]. The associated risk of developing obesity-related comorbidities earlier in life means that schoolchildren are a key target population for the promotion of sustainable healthy behaviors [5].

Interventions to promote healthy behaviors in children have largely focused on the school setting [6,7], with only 7% of randomized controlled trials (RCTs) targeted at the home setting [6]. The influence of parents and other family members on health behaviors in children is well established [8,9]. Research has highlighted the need for interventions that target children within the home environment [10], which encourages positive behaviors before children progress into adolescence and develop more autonomy over their health choices and the influence of the family context wanes [10]. Family-based interventions typically involve target children and at least one other family member, typically a parent [11]. Without the involvement of family members in interventions, long-term behavior change is unlikely to be sustained in children [12]. A recent meta-analysis identified 19 family-based interventions targeting physical activity, with 66% of included studies reporting a positive effect on physical activity [12]. This is in contrast to the lower levels of effect noted in reviews of school-based interventions [13,14]. Family-based interventions that target diet alongside physical activity appear to be more effective in reducing BMI z-score when compared with diet-only or physical activity–only interventions; however, the evidence is considered to be of low certainty [6]. Furthermore, interventions that target the family psychosocial environment and emphasize the child as the agent of change warrant further investigation [12].

Alongside family involvement, incorporating technology within the family setting has been identified as a potential means of enhancing the effectiveness of interventions targeting childhood obesity [15,16] and may also present further opportunities to increase the reach of interventions [16]. There has been a rapid increase in interventions adopting technology, as it can provide a cost-effective means of providing information and feedback alongside existing interventions or can function as a stand-alone intervention [17,18]. Children and adolescents have been described as *digital natives*, having been exposed to technology for most of their lives [19]. This coupled with high levels of smartphone ownership (78% of adults) and broadband connections (80% of homes) [20] highlights the potential of internet-based technologies for changing health behaviors.

Researchers and practitioners have used technology to change how we deliver interventions (eg, moving from print-based information to web-based resources) and how we incorporate behavior change techniques within interventions. To date, interventions using interactive electronic media [18] or web-based management programs [21] have demonstrated some potential for weight management; however, studies were generally of a lower quality and largely conducted in the United States [18]. A recent systematic review identified 8 eHealth interventions (comprising internet-based interventions, voice prompts, or telemedicine) whereby parents or guardians were the agents of change [16]. Included studies did not report a significant effect on BMI or BMI z-score; however, half of the interventions reviewed found significant improvements in physical activity–related or dietary-related outcome measures [16].

There is a strong need for research studies to target the family setting [22]. Innovative interventions are required [23], with the aim of improving both parents’ and children’s behaviors. In addition, there is a need for interventions to include more detailed process evaluation with their methodology to further understand the reasons why certain interventions are, or are not, effective in this setting [6]. Intelligent personal assistants (eg, Amazon Alexa) represent an efficient, low-cost method of delivering individualized behavioral interventions, with the potential for scaling at the population level [24]. Unlike other technologies such as wearable devices (pedometers, Fitbit, etc), which have been a primary focus for research studies in recent years, little is known about the potential role that intelligent personal assistants can play in positively influencing health-related behaviors [25].

This study (1) outlines the development of the GetAMoveOn+ Intelligent Personal Assistant Project (IPAP), (2) compares the acceptability of intelligent personal assistants alongside an existing intervention or as a stand-alone intervention, and (3) evaluates the potential of intelligent personal assistants for promoting and maintaining physical activity and other health-related behaviors in both parents and children.

## Methods

### Study Design

IPAP was a 12-week RCT conducted in 2 phases. Phase 1 was an RCT that evaluated the effect of a home-based intelligent personal assistant intervention on obesity-related behaviors (diet and physical activity) in families attending a community-based weight management project.

Phase 2 was an RCT that evaluated the effect of the home-based intelligent personal assistant in families not attending a weight management project. Randomization for both phases of recruitment took place at the family level, with families (a parent and 1 or 2 children) randomly allocated to an intervention or control group. Randomization was performed by a university staff member who was independent of the research team. Sealed, opaque envelopes were used to randomly assign families to a study arm.

### Participants

Families were eligible to participate when at least one child (aged 5-12 years) and one parent or adult responsible for their care consented to participate in the study. Given the nature of the intervention, access to internet connection with their home (Wi-Fi) and ownership of one smart device within the home (eg, a tablet or smartphone) or access to a computer or laptop to enable the family members to interact with the home-based intelligent personal assistant was required. The adult and child or children taking part in the study also had to live within the same household. No restrictions were placed on the family type. No inclusion criteria were placed on parents or children in relation to any medical condition. Participants were asked to notify the research team of any related issues that might affect participation in the intervention. No issues that limited or affected participation or resulted in adverse events were reported.

### Recruitment

#### Phase 1

All families (n=16) attending a community-based obesity prevention project, Safe Wellbeing Eating & Exercise Together (SWEET) as a family, were invited to participate in the study. The SWEET project is a community-based obesity prevention and management program aimed at children and families across a number of sites (community organizations, healthy living centers, etc) in the Western Trust area of Northern Ireland. It aims to work with families in areas of high economic deprivation and targets lifestyle characteristics, such as dietary habits, physical activity, and mental well-being. Families are recruited to the SWEET project via social media sites, flyer distributions in schools, and local paper advertisements. Before approaching families, permission was obtained from the Healthy Lifestyle Coordinator of the Healthy Living Centre where the project was being delivered. Members of the research team attended the first session of the project and provided a verbal overview of the research study. Written informed consent was obtained from all parents or guardians, and written parental consent and child assent were obtained for each child. Phase 1 of the study was conducted from January to April 2019.

#### Phase 2

Phase 2 was subsequently undertaken to further assess the acceptability of intelligent personal assistants as a stand-alone intervention. Potentially eligible families (as mentioned earlier) were invited to take part in the study (not restricted to those attending the SWEET project) through a number of recruitment strategies. Local community group leaders were contacted and asked to provide permission for a member of the research team to approach families (parents) at relevant events, for example, parent or child groups, youth club, sports training sessions etc. Similar to phase 1, prospective families were provided with a verbal overview of the study and detailed written information on the study. Written informed consent was obtained from all parents or guardians, and written parental consent and child assent were obtained for each child. Efforts were made by the research team to ensure families in phase 1 and phase 2 were recruited from similar community groups to avoid any potential sampling bias. Phase 2 of the study was conducted from May to August 2019. Families were only able to participate in one phase, that is, families who took part in phase 1 were not eligible to take part in phase 2.

### Intervention Selection

A smart speaker (Amazon Echo) and its linked intelligent personal assistant (Amazon Alexa) were chosen as the tools for intervention delivery in this study. A market survey (n=2274) highlighted that 33% of respondents based in the United States and the United Kingdom owned a smart speaker [26]. Among these, Amazon’s devices were the most popular.

Intelligent personal assistants can perform a range of basic home assistant functions, including playing music, setting alarms, checking the weather, and searching for information. Users can also personalize the devices by adding apps or *Skills*, which further the device’s capabilities [25]. Research has shown that *health and fitness* apps are readily available for devices, with health education and fitness training apps the most common types of *health and fitness* apps [25]. The IPAP intervention involved using the existing features and skills developed for Amazon Echo devices.

### Intervention Description and Protocol

Following the completion of baseline measurements, families recruited to both phase 1 and phase 2 of the study were randomly allocated to either the intervention group (receive an intelligent personal assistant) or the control group (continue as usual without the provision of additional technology within the home). The IPAP intervention aimed to promote positive health behaviors in the family setting through the utilization of the functions of a smart speaker and its linked intelligent personal assistant. Each family in the intervention arm of the study received a smart speaker (Echo Dot, third generation, Amazon 2018 release) for use in the family home for the duration of the intervention (12 weeks).

The research team set up an individual user account for each family, creating a new email and password, not linked to the family’s other email accounts (for security purposes). Each family was provided with their log-in detail, meaning that the research team and family members could both access the accounts during the intervention period. Each family was provided with a detailed information sheet on how to set up and use the device and were instructed to contact a member of the research team for support or troubleshooting throughout the intervention period.

The research team was able to remotely access the devices and set weekly tasks, prompts, and reminders for family members. The prompts and reminders provided by the research team were developed in line with recommendations for the management of childhood obesity [27] and based on current public health recommendations in relation to physical activity [28] and dietary habits [29]. Examples of weekly prompts or reminders and potential ways in which the family could interact with the device are shown in Table 1. For phase 1, the intervention content from the device was aligned to the topics covered at each week of the SWEET program, ensuring that the message was appropriate for the target population. Families received one specific reminder or prompt per day, which was repeated at a number of times throughout the day to maximize reach. Reminders or prompts were delivered in the morning (before work or school) and in the evening. Families were asked to advise the research team of the most convenient times to receive the prompts or reminders. Families were also encouraged to inform the research team if they were missing the prompts or reminders. In these instances, the timings were revised.

**Table 1:** Examples of intervention components delivered by the intelligent personal system.

Intervention component and type of interaction		Interaction content
**Diet**		
	Skill	Ask *Vitality* [device-based skill] to give you a recipe—pick a simple meal and have a go cooking with Alexa
	Task	Plan your shopping list for the week and add foods to your list using Alexa
	Tip	Fruit and vegetables that are fresh, frozen, or tinned all count toward your 5-a-day
	Reminder	How much water have you had today?
**Physical activity**		
	Skill	Use Alexa to find some fun games that can help you be active
	Task	Kids, do 10 star jumps every morning
	Tip	You should aim to be active daily—try going for a 30-min walk on most days this week
	Reminder	Have you been for a walk as a family this week?

In addition, families were informed that the devices were to be used as a health promotion tool within the home setting and were free to add their own reminders at times convenient to them and had complete autonomy over what *Skills* (apps) they wanted to enable on their devices. A specific *Skill* was not developed for this intervention; rather, families were signposted to search for *Skills* under the topics of Health and Fitness, Lifestyle, Sport, Cooking, and Recipes. Within this, families could choose the skills most suitable for their children’s age and interests. In addition to the preprogrammed messages controlled by the research team, families were instructed that they were free to use the devices for other general functions, not specific to the research project.

Families were informed during the recruitment and throughout the intervention that the research team would also be able to view and manage their user accounts. Families were also made aware that all interactions with the device would be noted by the research team, including interactions that may not be linked to the goals of the intervention, for example, asking the intelligent system nonrelated questions.

### Outcome Evaluation Measures

Within this pilot feasibility study, we aimed to evaluate the potential of intelligent personal assistants for promoting and maintaining physical activity and other health-related behaviors in both parents and children. Data collection was carried out at local community centers or at the university by trained researchers, and all participant outcome measures were assessed at baseline and follow-up (12 weeks).

#### Physical Activity

Physical activity was measured using an ActiGraph GT3 accelerometer (ActiGraph LLC). Participants (parent and child or children) were instructed to wear the device on the waist for 7 consecutive days, removing it only for bathing, water-based activities such as swimming, and when asleep. During the measurement periods, participants were asked to keep a family log of when they wore the accelerometer and took it off. A sampling epoch of 15 seconds was used for data collection. Periods of ≥60 minutes of zero counts were classified as nonwear and were removed. Cutpoints were used to estimate time spent in sedentary behavior and light-, moderate-, and vigorous-intensity physical activity for adults [30] and children [31]. The primary outcome was total physical activity (light, moderate, and vigorous physical activity combined). Secondary accelerometer outcomes included data provision and the proportion of participants meeting the recommended guidelines for physical activity [28]. Participants who provided at least three weekdays of at least 480 minutes of data between 5 AM and 11.59 PM were included in the analysis. Families were given an incentive at each time point for returning the devices (GBP £20 [US $27] One4All voucher).

#### Health Outcomes

Height (nearest 0.1 cm) and weight (nearest 0.1 kg) were measured according to standardized protocols. BMI was calculated and converted to BMI z-scores using the World Health Organization AnthroPlus software (version 1.0.4).

#### Family Eating and Activity Habits

Behaviors related to eating and activity habits were assessed using the Revised Family Eating and Activity Habits Questionnaire (FEAHQ-R). The FEAHQ-R is a 32 item self-report instrument designed to assess changes in eating and activity habits of family members as well as obesogenic factors in the overall home environment (stimulus and behavior patterns) related to energy balance [32]. The questionnaire was completed by one parent on behalf of themselves, their spouse, and their child. Summary scores were calculated for physical activity, eating style, stimulus exposure (eg, unhealthy snacks at home), and eating related to hunger. A reduction in scores signifies improvements across all domains.

### Process Evaluation

#### Device Interactions and Usage

The research team was able to access each family’s account via their log-in details and view each interaction with the device across the intervention period. An interaction was defined as any engagement with the device made by a parent or child, in addition to the reminders and information provided by the device from the research team. A copy of all interactions was downloaded from the device website and anonymously stored. The research team recorded the number of interactions and the type of interaction. Interactions were primarily coded as *relevant* (related to physical activity or diet or well-being) or *nonrelevant* (ie, not related to the intended purpose of the intervention), with relevant interactions further coded based on their theme. For example, “How many portions of fruit and vegetables per day should I eat?” was recorded as a relevant interaction and subcoded under *Healthy-eating question*. *Waking up* the device, controlling volume, and prompts such as *Next song* were not recorded as interactions for the purposes of this study. In instances where the device was not able to provide a transcript of the voice command received, the device registered this interaction as, “Text not found. Click here to listen to the recording.” The research team did not listen to the voice recordings or include these in the interaction analysis. It was not possible for the research team to distinguish whether a parent or child interacted with the device.

#### Intervention Acceptability

A record of any technical issues in relation to the smart speaker was held by the research team. All parents in the intervention arm of phase 1 and phase 2 were invited to participate in focus group discussions. These discussions focused on the acceptability of the intelligent personal assistants, intervention fidelity, any challenges that arose during the intervention, and suggestions for future improvements. Owing to practical issues (timing and location), it was not possible to facilitate focus groups with all parents, so these were replaced with semistructured interviews. One focus group (n=4 parents) and 3 semistructured interviews (n=3 parents) were conducted with participating parents in the intervention arm of the study. All discussions were audiorecorded. The mean duration of the recordings was 26 (SD 20) minutes.

### Ethical Considerations

Participants were provided with detailed instructions on the use of the device and the functionality of the device, that is, what the device is capable of doing and picking up. The mute or temporality disable functions of the device were also highlighted to families. These instructions were developed using the manufacturer’s instructions. As these devices were present within the home and accessible to both parents and children, a protocol was developed to consider the potential issue of disclosure and unintended collection of data. No such issues were observed during the intervention period. The search history of the device was kept confidential, and the device was not used for any other purpose during the intervention, for example, recording information or conversations within the home. This pilot feasibility study was approved by the Ulster University Research Ethics Committee and was registered retrospectively (ISRCTN16792534).

### Data Analysis

#### Quantitative Analysis

Frequencies, percentages, means, and SDs were used to describe data related to recruitment, retention, outcome measures, intervention acceptability, device interactions, and usage. Data analysis was conducted using SPSS for Windows (version 25; SPSS Inc).

#### Qualitative Analysis

Focus groups and semistructured interviews were transcribed verbatim and analyzed thematically, following a deductive approach [33]. Following familiarization with the data, each transcript was reviewed for meaningful quotes and systematically coded by a member of the research team. Potentially relevant codes were grouped together to develop themes, which were reviewed to ensure representativeness. These themes were then reviewed by a member of the research team to ensure that the themes were representative of the coded excerpts. Coding and reviewing of themes were repeated independently by a second member of the research team.

## Results

### Recruitment and Retention

#### Phase 1

**Figure 1:**
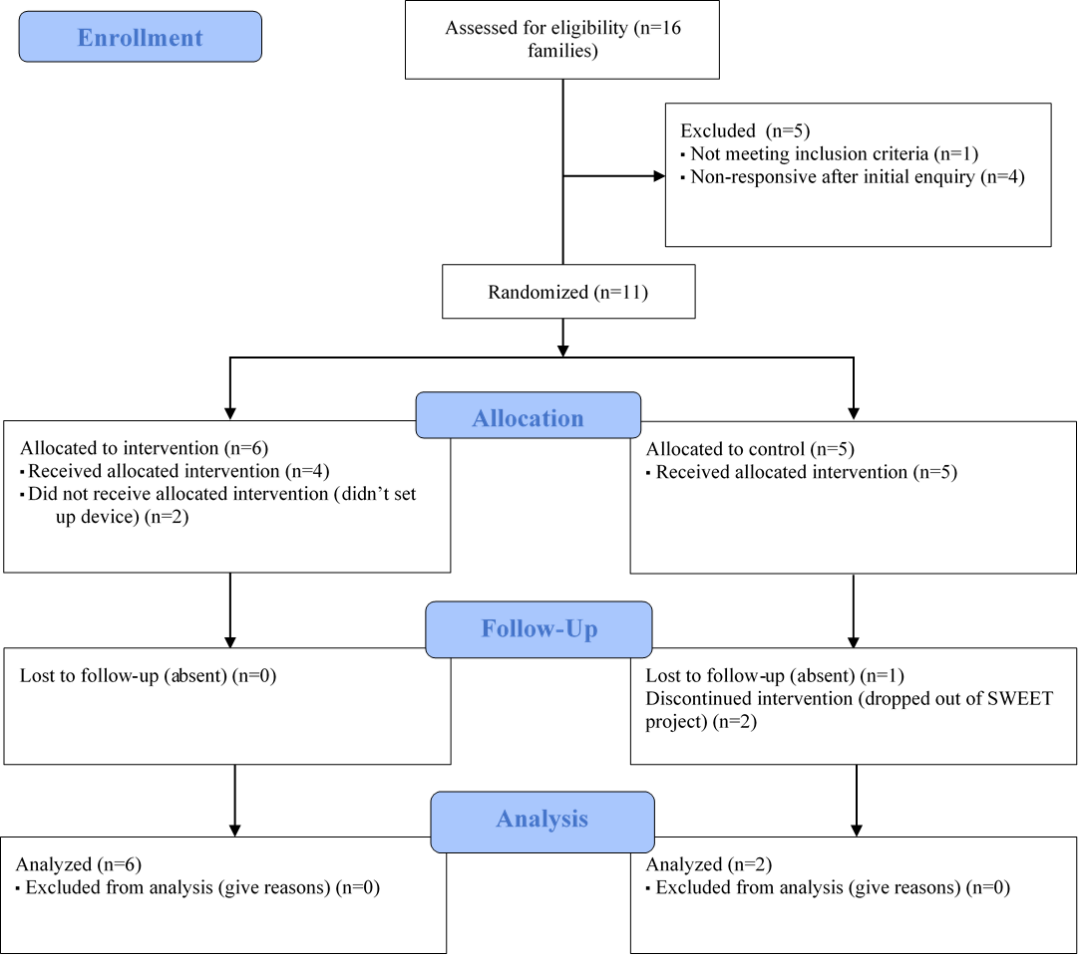
Consolidated Standards of Reporting Trials 2010 flow diagram for phase 1 participants. SWEET: Safe Wellbeing Eating & Exercise Together.

A total of 16 families attending the SWEET project were invited to participate in the IPAP study (Figure 1). Of the 16 families approached, one family was excluded for not meeting the inclusion criteria and 4 families failed to respond to the initial invitation. Of the 6 families allocated to the intervention, 2 families did not set up the device. Of those allocated to the control arm, one family was absent for follow-up measurements, whereas a further 2 families discontinued the SWEET project and subsequently this study as well. Participant characteristics are described in Table 2. All adult participants were categorized as overweight or obese at baseline.

**Table 2:** Individual participant characteristics at baseline.

Characteristic	Phase 1		Phase 2	
	Adults (n=11)	Children (n=16)	Adults (n=15)	Children (n=18)
Sex, female, n (%)	10 (91)	9 (56)	11 (73)	8 (44)
Age (years), mean (SD)	40.5 (5.4)	9.1 (2.0)	38.9 (5.2)	7.9 (2.0)
Height (cm), mean (SD)	166.0 (6.2)	141.1 (14.5)	166.9 (8.5)	130.0 (12.8)
Weight (kg), mean (SD)	97.0 (22.8)	49.5 (15.4)	81.4 (15.8)	28.3 (7.7)
BMI (kg/m^2^)	35.0 (6.4)	N/A^a^	29.1 (4.9)	N/A
BMI, z-score	N/A	2.61 (1.23)	N/A	0.02 (1.17)

^a^N/A: not applicable.

#### Phase 2

**Figure 2:**
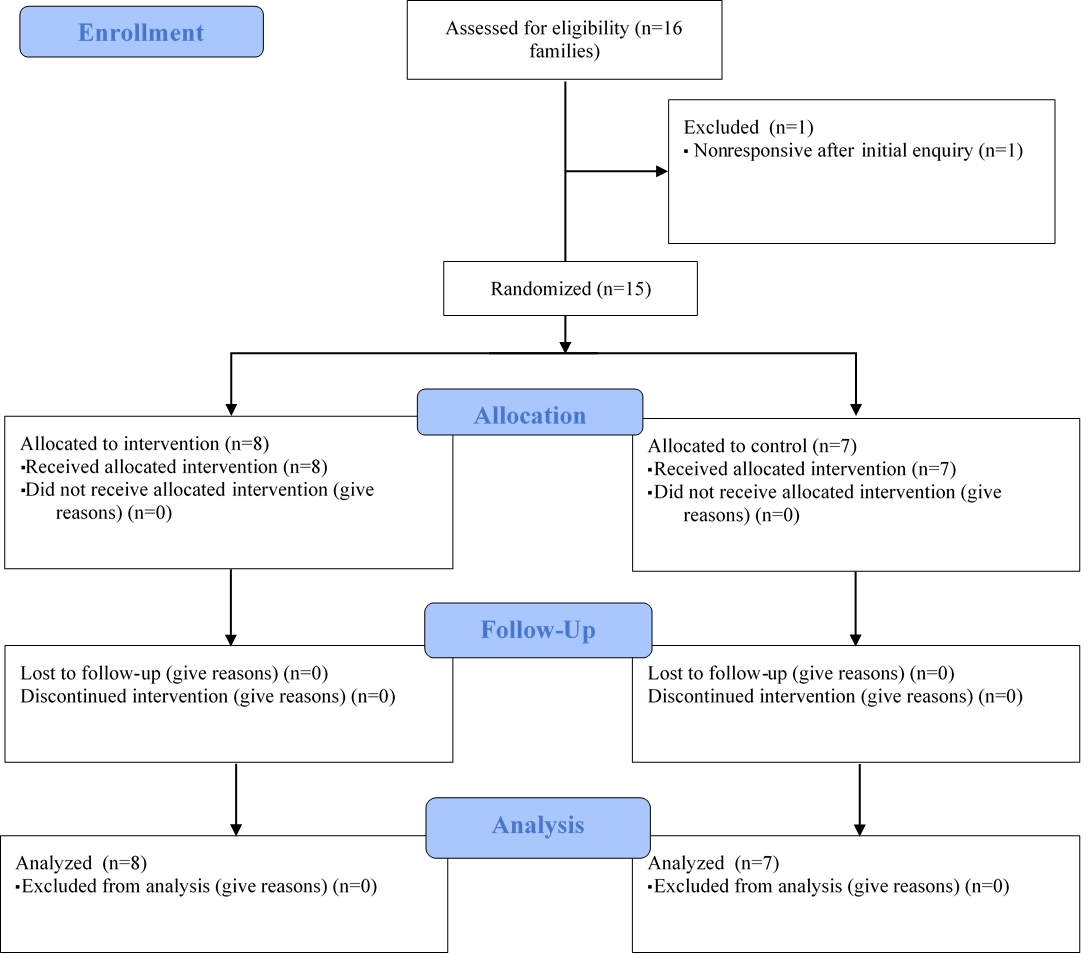
Consolidated Standards of Reporting Trials 2010 flow diagram for phase 2 participants.

A total of 20 families from local community groups were approached to take part, of which 16 were assessed for eligibility (Figure 2). Of 20 families, 15 were enrolled in the IPAP study, with all families retained at follow-up. Participant characteristics are described in Table 2. Overall, 80% (12/15) of adult participants were categorized as overweight or obese at baseline.

### Outcome Evaluation Measures

#### Physical Activity

In phase 1, 91% (10/11) of adults and 69% (11/16) of children met the minimum inclusion criteria for accelerometer wear time. At baseline, the mean valid wear time was 720 (SD 90.3) and 657.2 (SD 47.8) minutes per day for adults and children, respectively. At follow-up, the proportion of participants meeting the minimum inclusion wear time dropped to 55% (6/11) of adults and 19% (3/16) of children. In phase 2, 86% (13/15) of adults and 89% (16/18) of children met the minimum inclusion criteria for accelerometer wear time. At baseline, mean valid wear time was 782.1 (SD 63.2) and 695.4 (SD 36.3) minutes per day for adults and children, respectively. At follow-up, the proportion of participants meeting the minimum inclusion wear time remained at 87% (13/15) of adults and dropped to 72% (13/18) of children, indicating greater compliance to the accelerometer outcome measure in phase 2 of the IPAP study.

Of those who fulfilled the minimum wear time criteria, 70% (7/10) of adults and 36% (4/11) of children achieved the recommended physical activity guidelines at baseline for phase 1, compared with 77% (10/13) of adults and 38% (6/16) of children in phase 2 of the study. Owing to the small sample size, statistical testing was not undertaken to assess changes in physical activity before and after intervention (Table 3). Adherence to the accelerometer protocol may have been affected by the timing of the intervention and follow-up measurements coinciding with school holidays.

**Table 3:** Change in accelerometer measured physical activity and sedentary behavior across the Intelligent Personal Assistant Project study (adults).

Physical activity and sedentary behavior			Intervention, mean (SD)	Control, mean (SD)
**Phase 1**				
	**Baseline (n=10)**			
		Daily physical activity (minute per day)	268.5 (35.3)	234.2 (67.4)
		Sedentary behavior (minute per day)	440.5 (115.5)	492.8 (52.5)
	**Follow-up (n=6)**			
		Daily physical activity (minute per day)	293.7 (57.8)	201.1 (9.5)
		Sedentary behavior (minute per day)	587.6 (132.8)	531.4 (26.9)
**Phase 2**				
	**Baseline (n=14)**			
		Daily physical activity (minute per day)	260.7 (35.6)	241.8 (47.7)
		Sedentary behavior (minute per day)	562.3 (10.1)	492.7 (56.6)
	**Follow-up (n=12)**			
		Daily physical activity (minute per day)	218.9 (40.7)	244.8 (33.1)
		Sedentary behavior (minute per day)	513.9 (65.1)	498.3 (21.4)

#### Family Eating and Activity Habits

Questionnaire data were provided by 85% (22/26) of adult participants at all time points. In phase 1, positive improvements in scores for eating style were observed for adults (−1.75, SD 2.06) and children (−0.50, SD 2.81) in the intervention group, with increases observed in the control group. In phase 2, there was a slight improvement in both the activity level score and stimulus exposure and control for children in the intervention group, with all other summary scores increasing across the intervention period (Table 4).

**Table 4:** Change in scores for Family Eating and Activity Habits Questionnaire for adults and children in phase 2.

Characteristics	Adults, mean (SD)		Children, mean (SD)	
	Intervention (n=6)	Control (n=5)	Intervention (n=7)	Control (n=4)
Activity level	0.75 (2.72)	1.70 (2.11)	−1.07 (8.23)	−0.25 (6.65)
Eating style	1.80 (8.56)	5.33 (1.15)	3.33 (6.65)	−1.00 (3.00)
Eating related to internal cues	0.83 (1.33)	0.00 (2.00)	1.14 (1.46)	−0.13 (1.55)
Stimulus exposure and control	1.80 (4.09)	1.25 (4.99)	−0.25 (6.65)	0.00 (4.63)

### Process Evaluation

#### Device Interactions and Usage

Across phase 1 of the intervention, families who received a smart speaker on average interacted with the intelligent personal assistant (Alexa) 65 times. *Waking up* the device, controlling volume, and prompts such as *Next song* were not recorded as interactions for the purposes of this study. *Other* (including general knowledge questions and jokes) and *Music* were the most frequently observed interactions across the intervention period. Overall, 42% of all device interactions were coded as relevant in phase 1 (ie, related to diet, physical activity, or well-being). Reminders or prompts involved the family setting their own reminders. Examples of *Skills (diet)* and *Skills (physical activity)* used by families across the intervention period included fitness skills, recipe skills, and active game skills. During phase 1, the prompts or reminders provided by the research team aligned with the topics and tasks the families were covering in the SWEET project.

In phase 2, families did not attend the SWEET project, but the intervention content largely reflected the prompts or reminders provided to families in phase 1. Device interactions across phase 2 of the intervention were much higher, with families interacting with the device 312 times across the intervention period (equivalent to 31.3 interactions per week). Only 11% of interactions were coded as relevant (related to diet, physical activity, or well-being). Of the interactions that were coded as relevant, the most frequent interactions were when families asked questions about nutrition (healthy eating) or used *Skills* related to healthy eating, for example, recipes or healthy eating tips.

#### Intervention Acceptability

In total, 7 parents took part in focus groups and semistructured interviews to discuss their experiences of the IPAP project. At the offset of these discussions, parents acknowledged the prominent role of technology in their family’s everyday lives and the need to use it in a positive way:

Technology is there, and it can be used for good and evil. And it’s not going to go away. The way they are growing up, they can’t avoid it really so might as well try and use it for good.

Family 4, male

...*they are probably more motivated by it [technology], so it probably is the future for the younger generation*...

Family 6, female

Parents commented that the intelligent personal system motivated the child to engage with the intervention:

It actually motivated her quite a bit, because she was saying “mummy, we need to go for a family walk now...or I need to eat my fruit or...”

Family 6, female

Families found the intervention content acceptable and discussed how the prompts or reminders encouraged them to change their behaviors in a fun way (Table 5). Families also highlighted how they used other features, such as the skills for recipes or home workouts (Table 5).

Families highlighted several ways to increase engagement with the intervention, including further suggestions on how to use the device within the home and more personalization of the prompts or reminders. The timing of prompts or reminders was a key component of the intervention delivery, and families noted practical issues with this, in addition to the importance of ensuring that families were at home when the device was interacting with them (Table 5). Parents suggested incorporating other technologies alongside the intelligent personal system to facilitate this:

If it was connected to your phone, like a phone reminder as well, because Alexa’s in the house.

Parent 2, female

In addition, families felt that the device needed to be linked to some type of feedback to increase accountability and provide families with opportunities to log their healthy eating or physical activity (Table 5).

Parents felt that the intelligent personal assistants played an additive role in encouraging children to be healthier and could work alongside other types of intervention:

I still think you need the traditional ways of activity rather than reliance on a device.

Family 6, female

...if there was an intervention or like, if there were a, a class or some sort of, erm, programme that was with, sent home with families and Alexa reminded you to do it...

Family 6, female

In terms of concerns about having a smart speaker within the home, most parents commented that they were cautious of both increasing engagement with technology and the potential issues with social media and young people (Table 5). These concerns regarding internet access or social media were more prominent from parents than issues specific to the intelligent personal assistants themselves:

...he’s downloading games and I don’t know what they are—I would be quite worried; not so much that it’s listening, I wouldn’t worry about Alexa listening, it’s not gonna hear anything in my house.

Parent 7, female

**Table 5:** Supporting quotes from family focus groups and semistructured interviews.

Subheading		Supporting quotes
**Findings related to intervention delivery**		
	Device setup	“It was easy to set up and easy to use. Quite interesting but, and the prompts were very good*.*” [Family 1, female]
	Prompts or reminders from the research team	“We got a prompt, quick do 10 sit ups, and I’m like come on children, everyone on the floor, let’s do it! It was some craic [fun] like, and everybody just downed the phones and going to do that challenge. They loved it.” [Family 3, female] *“*The whole jist of it was brilliant, like the wee prompts it tells you...try this or try that, you know it’s just planting that wee seed in your head and when that wee seed’s planted, obviously you are gonna try aren’t you, so I think it is a great thing.” [Family 2, female]
	Using other device features	“...the easy access to the workouts so that you could just do it at a time whenever it suited you*.*” [Family 6, female] “There were a couple of occasions where we asked Alexa for a healthy recipe to make something so we made a chilli one day and we asked Alexa for a recipe ‘cos we were prompted by the device about, you know, healthy, eating healthily and stuff*...*” [Family 5, male] “[Child name] was new-fangled with it, she was more into the music in it, bopping about but it got her active too, she was asking me how to do this, and will you do this *‘*Flossing’...it was good from that point of view you know.” [Family 2, female] “Even her homeworks, she was going out and asking, she was asking me how to spell this, I said ask Alexa, just to get her doing things for herself.” [Family 3, male]
	Overall device usage	“We probably could have utilised it much more but it’s just the pure fact if we had more time. Erm, and the fact that we were away from it all day long and then we came in, in the evening, it’s usually kind of a race, get the dinner made and...” [Family 6, female] “...but after, like, a week or so they kind of almost forgot it was there and maybe that was our fault, we didn’t encourage them to use it as much, erm, but the prompts I think were a good idea.” [Family 5, male]
**Findings related to intervention optimization**		
	Timing of prompts or reminders	“I think there was a couple of technical glitches where the timing wasn’t right because we didn’t seem to get the prompts and we used Alexa a lot like, we do ask a lot of questions and stuff but, erm, it didn’t seem to prompt us; maybe we were out at the time.” [Family 4, male] “You know, if we weren’t at home..., I don’t know how many prompts there were.” [Family 7, female]
	Lack of feedback provided	“...but what it would say to me, ‘Have you had your five a day?’ Do I shout back, ‘Yes,’ or, ‘Alexa, yeah,’ I don’t know what way to answer...” [Family 7, female] “If you had to log what you did, you know, because it’s fair enough, erm, you could say, ‘Right, go for a family walk,’ but you know, then they come back and say, ‘Well how many kilometres did you do?’ or whatever...to close the loop.*”* [Family 6, female]
	Concerns	“I just worry about that whole side of technology, erm, never mind Alexa but all social media, erm, in terms of how, how that can be utilised against them and I suppose that’s a worrying thing for me as a parent...” [Family 6, female] “I think if you find the right balance where, you know, I don’t like the idea of my kids being constantly engaged to technology but I can see the benefit of, of that via a prompt or something like that but, you know, I wouldn’t want them to be constantly going to Alexa...” [Family 3, male]
	Increasing device usage	“You know, I think they would maybe be set challenges to do because I think if they’ve, just can get an app and do so much, I’m not sure that they’ll benefit from it*.*” [Family 6, female] “I think if it was maybe a wee bit more personalised...I don’t actually know what I was supposed to be doing with Alexa, you know...and maybe it was in the documentation somewhere, maybe there was a letter written somewhere that I didn’t see, that I didn’t read.” [Family 7, female]

### Practical Considerations

Most families were able to set up their user accounts and link these to the smart speaker device. Overall, 2 families did not set up their devices in phase 1 of the study. Of these families, one parent noted that they could not set up the device because they shared the house with another family who did not want the device used, and the other family failed to respond to follow-up instructions from the research team, meaning they did not receive the intervention content. All families in phase 2 successfully set up and used the device.

The smart speakers had to be *online* to allow the research team to set up reminders or prompts and refresh information on the family’s interactions with the device. The 2 families in phase 2 had their devices set to *Offline* for extended periods, limiting the volume of interaction managed by the research team. A further family in phase 2 registered the device with their own personal Amazon account for 2 weeks during the intervention period; therefore, the research team was unable to set prompts or reminders or access information on the family’s interactions with the device over this period. A protocol was also put in place to cover the potential issue of disclosure of information and unintended collection of data; however, no scenarios arose within this study.

## Discussion

### Principal Findings

To our knowledge, this is the first study to outline the development and usage of intelligent personal assistants to promote positive health-related behaviors within the home setting. Given the constraints that exist within current family-based interventions, including time and travel restraints [10], moving toward novel interventions that incorporate web-based learning may help improve engagement and attrition [10]. Within this pilot feasibility study, we assessed the acceptability and feasibility of using intelligent personal assistants alongside more traditional intervention approaches or as a stand-alone intervention tool. This feasibility study demonstrated that using intelligent personal assistants to deliver health-related messages and information within the home was feasible, with high levels of engagement from participating families. This work also highlighted methodological considerations and opportunities for intervention improvement moving forward.

To date, there is a paucity of research on both the development of interventions using this technology and the potential effectiveness of such interventions. An ongoing study is examining the role of a voice coach intervention (Amazon Alexa/Echo) on increasing levels of physical activity among overweight and obese cancer survivors [24]. In addition, Public Health England has used intelligent personal assistants (Amazon Echo) to encourage parents to adopt healthy behaviors around breastfeeding [34] by providing parents with general information and tailored advice based on the age of their child. This study highlighted for the first time that families found this type of intervention approach acceptable and feasible within the home setting. Most families assigned to the intervention were able to set up and initialize their devices and engage with the intelligent personal assistant across the intervention period. Focus groups and interviews with parents highlighted that the prompts or reminders were particularly useful and commented that the intervention encouraged the family to be healthier in a fun way.

Recent research has highlighted the plethora of *Health and Fitness*–related apps available for smart speakers [25], with health education, fitness and training, and nutrition the most frequently occurring of these apps. For the purposes of the IPAP intervention, prompts or reminders provided by the research team were based on the devices’ existing functionality, and families were instructed to use the features already developed for these smart speakers. High levels of interaction were observed across the intervention period, with a higher volume of interactions in phase 2. Setting reminders or prompts, asking questions about nutrition, and using physical activity and nutrition apps (Skills) were the most common relevant interactions across the intervention period.

The mean frequency of device interactions across phase 2 was much greater (312 vs 65), but a higher proportion of interactions were coded as *relevant* in phase 1 (42% vs 11%). This provides important insights into how families used the devices and suggests that linking the devices to an ongoing intervention, as with phase 1, may be more directive in terms of prompting families to use the device for health-related interactions. The issue of families not adequately implementing intervention components has been highlighted in similar feasibility work evaluating the use of a web-based intervention to encourage families to increase their physical activity [23]. Within this study, families were provided with written instructions and reminders on how to interact and engage with the intelligent personal assistants. Parents highlighted several ways to improve engagement with the intervention, including incorporating challenges, providing feedback, and clearer guidance from the intervention facilitators on how to use the device within the home. Within this feasibility study, the intervention facilitators were members of the research team. Given the important role of facilitators in terms of intervention outcomes [35], providing families with more guidance and training before the intervention, and ongoing support during the intervention, may improve the family’s utilization of the device [23].

Given the small sample size in this study, it was not possible to statistically compare the effectiveness of these 2 intervention approaches. As the families in phase 1 were already attending the SWEET project, the results from phase 1 and phase 2 could not be combined. A recent systematic review highlighted that most family-based eHealth interventions combined technology with other types of delivery, for example, face-to-face counseling, nutrition lessons, and so on, and from this literature, it is difficult to ascertain the exact effect of the eHealth component versus other approaches [16].

The development and feasibility testing of the intervention identified several important methodological considerations. First, the research team was not able to control the content, or indeed validity, of the responses families received when they asked for information on healthy eating or physical activity. At present, there is limited insight on whether these apps are developed based on evidence-based guidelines or available materials [25]; therefore, assessing the accuracy of educational information provided by these devices would be an important methodological consideration moving forward. Indeed, a previous study examining the provision of medical advice from these devices highlights the importance of cautioning users not to use such technologies in place of medical advice without consulting with their health care provider first [36]. Second, families noted that the intervention in its current format did not provide any opportunities for feedback or accountability with limited options for families to log their healthy eating or physical activity. Moving forward, studies should explore the potential of linking these intelligent personal assistants with other technologies to monitor behaviors, set goals, and provide feedback [37,38], which may help improve the effectiveness of technology-based interventions [39].

The implementation of the intervention was dependent on a few factors. An important practical consideration was the capacity of the research team to access the family’s device remotely. If the device was switched off or the family had Wi-Fi connection issues, the delivery of the intervention was affected, as the research team was unable to set new reminders and prompts during these periods. During the focus group and interview discussions, parents highlighted how the timing of the prompts or reminders may have affected their adherence to the intervention. Although attempts were made to tailor the intervention to suit the schedules of individual families, future studies using similar intervention components should seek to provide families with further guidance and ownership in relation to managing the devices themselves.

### Strengths and Limitations

The IPAP study adopted a cross-sectoral, interdisciplinary approach to explore the role of intelligent personal assistants within the home environment to promote and maintain physical activity and other health-related behaviors in families. The intervention development and evaluation used novel methods to capture intervention engagement, addressing key recommendations for research in this field to adopt appropriate methodologies that enable interventions to be effectively evaluated [17]. This study developed the intervention content and tested its feasibility in line with the best practice for intervention development [40]. Owing to the small sample size, no statistical analysis was undertaken at this stage to evaluate the effectiveness of the intervention. Accelerometer compliance was low during phase 1 of the study, despite the use of incentives to encourage adherence. In addition, device usage was much lower across phase 1. Given that these families were already taking part in the SWEET project at the time, they may have felt overburdened with data collection.

### Conclusions

This study demonstrates the feasibility and acceptability of a family-based intervention using intelligent personal assistants. This novel intervention has highlighted important methodological considerations and provides important suggestions to further optimize the potential of intelligent personal assistants to promote positive health-related behaviors in the home setting. This work will inform future pilot and fully powered studies to build upon this feasibility work and test whether such interventions are effective at changing health-related behaviors, including physical activity and healthy eating.

## Appendix

Multimedia Appendix 1 CONSORT EHEALTH checklist V1.6.1.

## Abbreviations

FEAHQ-R: Revised Family Eating and Activity Habits Questionnaire
IPAP: Intelligent Personal Assistant Project
RCT: randomized controlled trial
SWEET: Safe Wellbeing Eating & Exercise Together
